# Retraction: MicroRNA-219-2-3p Functions as a Tumor Suppressor in Gastric Cancer and Is Regulated by DNA Methylation

**DOI:** 10.1371/journal.pone.0282980

**Published:** 2023-03-29

**Authors:** The PLOS ONE Editors

The *PLOS ONE* Editors retract this article [1] because it was identified as one of a series of submissions for which we have concerns about peer review, authorship, and similarities across articles. Image similarities were noted between Fig 2C and Fig 3B in [1] and Fig 2C and Fig 3B in [2] respectively. These concerns call into question the validity and provenance of the reported results. We regret that the issues were not addressed prior to the article’s publication.

YM, CL, and JY agreed with the retraction. HL, ZL, ML, and YL disagreed with the retraction. DZ, LD, BW, HY, FW, and JZ either did not respond directly or could not be reached.
